# Extraction and purification of glomalin-related soil protein (GRSP) to determine the associated trace metal(loid)s

**DOI:** 10.1016/j.mex.2022.101670

**Published:** 2022-03-18

**Authors:** Hanyi Li, Bo Yuan, Chongling Yan, Qingxian Lin, Jiajia Wu, Qiang Wang, Jingchun Liu, Haoliang Lu, Heng Zhu, Hualong Hong

**Affiliations:** a Key Laboratory of the Ministry of Education for Coastal and Wetland Ecosystems, Xiamen University, Xiamen 361102, China; b State Key Laboratory of Marine Environmental Science, Xiamen University, Xiamen 361102, China; c State Key Laboratory of Grassland Agro-ecosystems, College of Pastoral Agricultural Science and Technology, Lanzhou University, Lanzhou 730020, China

**Keywords:** Metal speciation, Recalcitrant organic carbon, Natural organic matter, Organo–ferrihydrite co-precipitates

## Abstract

Glomalin-related soil protein (GRSP) is recalcitrant compound in the soil and sediment and plays a crucial role in metal transportation. However, potential metal speciation changes during GRSP extraction remain unreported. Here, a feasible GRSP extraction and purification procotol is described for robust determination of GRSP-bound metal(loid)s. Several spectrum patterns measured before and after GRSP extraction indicate that the GRSP extraction process does not significantly affect the mineral state of the samples. Potential bias generated by simultaneous metal release during GRSP extraction can be effectively eliminated by applying complete and independent dialysis.

• Na signal appeared in the X-ray photoelectron survey spectrum after GRSP extraction, suggesting that Na exchange may be a critical process in releasing metal(loid)s.
• Element maps obtained using secondary ion mass spectroscopy exhibited different distribution of C-N and Fe after GRSP extraction, thus suggesting that uncoupling of the Fe-organic framework occurred during GRSP extraction, which could result in the release of organic matter and metal(loid)s.
• European Community Bureau of Reference (BCR) subsequent extraction reveals that most of the metal(loid)s were extracted from the acid-exchangeable and residual fraction during GRSP extraction. Remarkable differences in the GRSP-bound metal content before and after dialysis implied that the dialysis could remove most metal(loid)s.

## Graphical abstract

**Figure:**
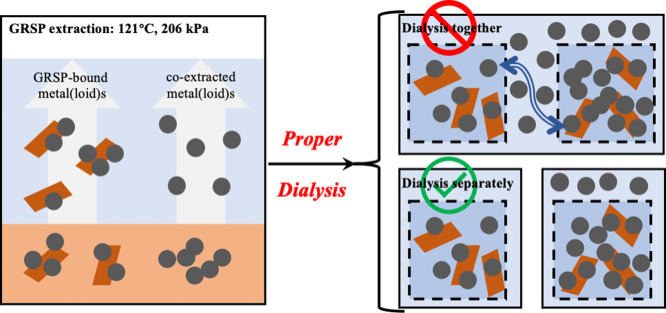

Specifications table

**Table:** 

Subject area:	Environmental science
More specific subject area:	Sample pretreatment step for analysis of GRSP-bound metal(loid)s
Method name:	Extraction and purification of GRSP to determine GRSP-bound trace metal(loid)s in sediment and soil
Name and reference of original method:	This protocol is based on guidance described in (Wright and Upadhyaya, 1996).
Resource availability:	N/A

## Method details

### Background and sample information

Glomalin is a recalcitrant glycoprotein produced by mycorrhizal fungi buried in soil, commonly defined as glomalin-related soil protein (GRSP) in geochemistry [1]. GRSP has been widely used in recent years to study metal pollution [2], [3], [4]. However, the metal speciation may change during GRSP extraction, and the simultaneous extraction of certain metal(loid)s may interfere with the accurate determination of GRSP-bound metal contents. A reliable sample pretreatment protocol is critical for studying the flux, enrichment, and bioaccessibility of GRSP-bound metal(loid)s.

Five samples (Table S1), three provided by the International Humic Substances Society (IHSS) and two collected in the field, were used to represent realistic complications encountered in actual field studies. Three IHSS reference samples (2BS103P, BS102M, BS104L) represented different sample types (peat, soil, and leonardite) with differing ranges of C, N, and Fe contents (C: 2.09%–42.8%, N: 0.25%–2.98%, Fe: 0.53%–2.22%). Two field samples (CSS1-36, CSS1-46) were collected to represent coastal sediment samples with higher salinity (Carter et al., 2009) and are therefore ideally suited for addressing questions regarding metal speciation during GRSP extraction.

### GRSP extraction

GRSP extraction was performed on each sample using a method slightly modified from Wright and Upadhyaya [3,5]. Briefly, 8 mL of sodium citrate buffer (50 mM, pH 8.0) was added to about 1 g of sample, and then the solution was placed in an autoclave (121 °C, 60 min) for repeated extractions until the supernatant lost its typical red-brown color. Between each cycle, the supernatant was collected by centrifugation (8,000 × *g*, 10 min). Before measurement, the mixed supernatant was centrifuged again (10,000 × *g*, 10 min), and the protein concentration in the extract was determined by Bradford analysis using bovine serum albumin as a standard.

### GRSP purification for metal content determination

The GRSP extracts were precipitated at a pH of 2.5 with 1 M HCl, incubated under ice-bath for 12 h, and then immediately centrifuged at 8000 × *g* for 10 min. The precipitate was redissolved entirely in 0.1 M NaOH, transferred to a dialysis tube (Dialysis molecular weight = 8,000–14,000 Da), and dialyzed against deionized water for 72 h. The samples were dialyzed separately, and the deionized water was changed every 8 h.

### Soil/sediment characteristics before and after GRSP extraction

**Fig. 1:**
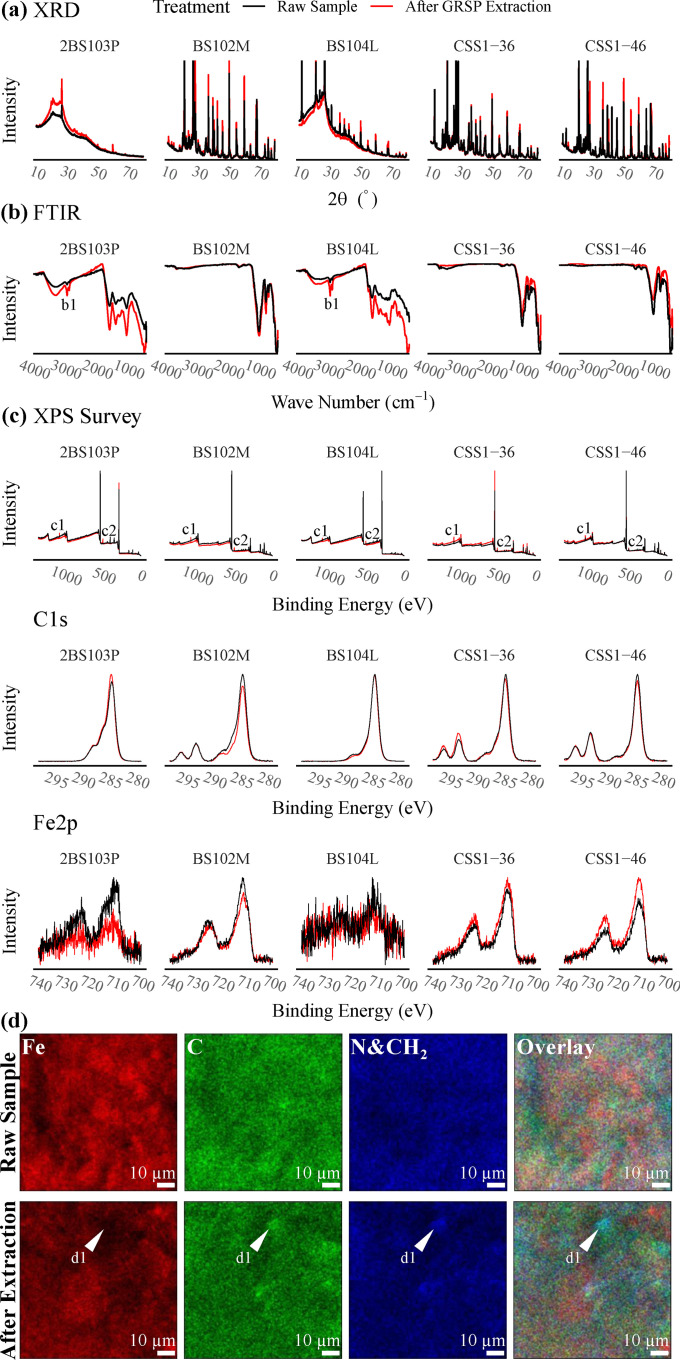
**Overlapping spectra comparing the representative samples before and after GRSP extraction.** 2BS103P–Pahokee (Florida) peat soil, BS102M–Elliott silt loam soil, BS104L–Gascoyne leonardite, CSS1-36&46–Danshuei River sediment. (a) XRD patterns; (b) FTIR spectra; (c) XPS patterns; and (d) SIMS element maps of sample BS102M.

The X-ray diffractometry (XRD) analysis reveals that the mineral phases in the samples were similar before and after GRSP extraction (Fig. 1a). The narrow peak widths and positions did not change after the GRSP extraction for each sample, although some peak heights showed a more noticeable difference in sample 2BS103P. This indicates that the mineral composition did not significantly change after GRSP extraction.

The Fourier transform infrared spectroscopy (FTIR) analysis (Fig. 1b) shows that the sediments had similar IR band frequencies and peak distribution line shapes before and after GRSP extraction. Elevated absorption of peak b1 was found in two samples, 2BS103P and BS104L. Bands at 2920 cm^−1^ and 2850 cm^−1^ of peak b1 was assigned to CH stretching in methyl and methylene groups. However, the IR absorption in the range of wave number 1500-500 cm^−1^ was also generally enhanced in these two samples, so we infer that this could be a result of the surface modification during the GRSP extraction. This indicates that the functional groups contained in the sediments were not significantly affected by GRSP extraction.

The X-ray photoelectron spectroscopy (XPS) survey spectra exhibit a clear Na signal (Fig. 1c, arrow c1, Na1s, arrow c2: Na auger), which suggests that Na substitution occurred during the GRSP extraction process that may have resulted in the release of a large number of metal(loid)s. The XPS spectra of C1s, N1s, and Fe2p exhibit the same peak distribution (Fig. 1c), except for a substantial decrease in the peak height in some samples, which indicates no significant valence change of C, N, or Fe during GRSP extraction.

Secondary ion mass spectroscopy (SIMS) showed the different distribution patterns of C, N (with -CH2), and Fe in BS102M before and after GRSP extraction (Fig. 1d). The three elements can be coupled at the microscopic scale in raw sample. However, C+N and Fe decoupling occurred (Fig. 1d, arrow d1) after GRSP extraction, indicating the coupling of carbon and iron is disrupted during the extraction of GRSP. The local Fe:C molar ratio affects this organo–ferrihydrite co-precipitates process [6,7]. Therefore, when the local Fe and C+N coupling distribution was lost, some heavy metal(loid)s immobilized via organo-ferrihydrite co-precipitates may be released into the solution. During this process, the metal speciation may change, resulting in the simultaneous release of metal(loid)s and bias on the determination of GRSP-bound metal(loid)s.

### Metal speciation before and after GRSP extraction

To determine the total contents of the investigated metal(loid)s in the raw samples, 0.1 g of the samples were placed in a closed container and, 5 mL of HNO_3_, 2 mL of HClO_4,_ and 1 mL of HF were then sequentially added, sealed, and left overnight. The closed vessel was then placed in an oven and digested in two steps (first step: 140 °C, 1 h; second step: 180 °C, 5 h). To analyze the content of the investigated metal(loid)s in the extracted GRSP, 1 mL of dialyzed or undialyzed GRSP sample was digested with 5 mL of HNO_3_. Inductively coupled plasma mass spectrometry (NexION 2000B, PerkinElmer, USA) was performed to determine As, Cd, Co, Cr, Cu, Fe, Ni, Mn, Pb, and Zn in the sequential extract and digested solutions.

To determine the metal speciation before and after extraction, we used a modified Community Bureau of Reference (BCR) sequential extraction process to extract the samples following the procedure described in Luo and Christie [8,9]. Briefly, 1 g of sample was placed in a 50 mL centrifuge tube, mixed with 20 mL of 0.11 M acetic acid, shaken for 16 h (overnight), and centrifuged at 5000 × *g* for 10 min. The supernatant was then collected to obtain the acid-soluble fraction of the metal(loid)s (M_ac_). The residue was subsequently mixed with 20 mL of 0.1 M pH 2.0 hydroxylammonium hydrochloride, shaken for 16 h, centrifuged at 5000 × *g* for 10 min, and the supernatant was collected to obtain the metal(loid)s as the reducible fraction (M_red_). The residue was oxidized by adding 10 mL of 30% hydrogen peroxide twice in a water bath at 85 °C. The residue was then added with 25 mL of 1 M pH 5.0 ammonium acetate buffer, shaken for 16 h, centrifuged at 5000 × *g* for 10 min, and the supernatant was collected to obtain the oxidizable fraction (M_ox_). The raw sample's residual fraction (M_res_) was calculated as the difference between the total metal content and the sum of the BCR-extractable metal(loid)s. After extraction, the residual fraction of the sample was calculated as the difference between the total metal(loid)s content and the sum of the BCR-extractable and released metal(loid)s during GRSP extraction. Inductively coupled plasma mass spectrometry (NexION 2000B, PerkinElmer, USA) was performed to determine As, Cd, Co, Cr, Cu, Fe, Ni, Mn, Pb, and Zn in the sequential extract and digested solutions.

**Fig. 2:**
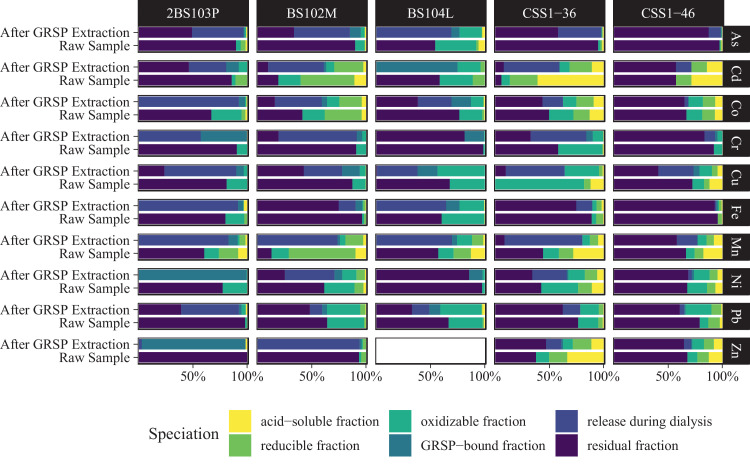
**Speciation of trace metal(loid)s in the representative samples before and after GRSP extraction.** The subfigures exhibited the speciated of As, Cd, Co, Cr, Cu, Fe, Mn, Pb, and Zn in the raw samples and samples after GRSP extraction. 2BS103P–Pahokee (Florida) peat soil, BS102M–Elliott silt loam soil, BS104L–Gascoyne leonardite, CSS1-36&46–Danshuei River sediment.

A large proportion of M_ac_, M_red,_ and M_ox_ could be released when these fractions were relatively high (Fig. 2, *e.g.,* Cd_ac_ in CSS1-36; Co_red_ in BS102M; Cu_ox_ in 2BS103P), which suggests that the GRSP-bound metal(loid)s had multiple sources, which is consistent with the characteristics of their iron-containing glycoprotein complexes. However, large amounts of M_res_ metal(loid)s were still be released during the extraction process when the sediment was dominated by M_res_, which suggests severe interference by the co-extracted metal(loid)s.

### Avoiding co-extraction bias in GRSP-bound metal determination using proper dialysis

The metal(loid)s in the dialyzed GRSP extract only accounts for a tiny fraction of the undialyzed extract (Fig. 2). This indicates that metal(loid)s can be removed during dialysis, thus leaving the metal(loid)s firmly bound to GRSP, which is more similar to the immobilization of heavy metal(loid)s by GRSP under natural conditions. The undialyzed samples contain exceedingly high levels of synchronously extracted metal(loid)s and are thus not recommended for direct metal determination.

**Fig. 3:**
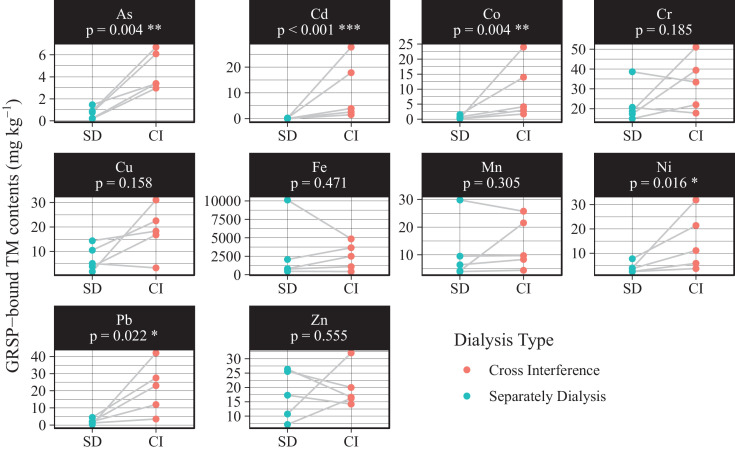
**Separate dialysis avoids metal interferences between samples compared to mixed dialysis.** Paired t-tests were used to detect significant differences in metal(loid) contents under different dialysis methods

Moreover, the dialysed GRSP was placed in a solution with 0.5 μg/L metals (As, Cd, Co, Cr, Cu, Fe, Ni, Mn, Pb, and Zn) to simulate the cross-interference caused by the release of high concentrations of heavy metal(loid)s during mixed dialysis. These spiked GRSP samples were subsequently dialyzed separately at the same time as the normal samples. Even after adequate dialysis, the spiked samples still exhibited cross interference: As (t = -6.1, *p* = 0.004), Cd (t = -20.5, *p* < 0.001), Co (t = -5.8, *p* = 0.004), Ni (t = -4.0, *p* = 0.016), and Pb (t = -3.6, *p* = 0.022) were significantly elevated in the spiked samples (Fig. 3). The critical role of dialysis for robust GRSP-bound metal determination should be emphasized, which should be noted in subsequent studies.

## Conclusion

This study demonstrates the potential interference of co-extracted metal(loid)s during GRSP extraction and the methods to mitigate this impact. Our results indicated that the simultaneous release of metal(loid)s during GRSP extraction can introduce bias in determining GRSP-bound metal(loid)s contents. Na exchange and decoupling of organo–ferrihydrite co-precipitates is cretical for metal(loid) release. The large amount of metal(loid)s released from M_res_ interferes with the robust determination of GRSP-bound metal contents. Independent dialysis can very effectively remove the bias from the simultaneous extraction of metal(loid)s. Therefore, independent dialysis was recommended as a mandatory step in subsequent studies on GRSP-bound metal(loid)s.

## CRediT authorship contribution statement

**Hanyi Li:** Conceptualization, Formal analysis, Writing – original draft. **Bo Yuan:** Methodology, Writing – original draft. **Chongling Yan:** Conceptualization, Funding acquisition, Writing – review & editing, Supervision. **Qingxian Lin:** Investigation, Writing – review & editing. **Jiajia Wu:** Formal analysis. **Qiang Wang:** Methodology, Resources. **Jingchun Liu:** Resources, Project administration. **Haoliang Lu:** Methodology, Resources. **Heng Zhu:** Funding acquisition. **Hualong Hong:** Conceptualization, Writing – review & editing, Data curation.

## Appendix A Supplementary materials

Supplementary Data S1 Supplementary Raw Research Data. This is open data under the CC BY license http://creativecommons.org/licenses/by/4.0/ Supplementary Data S1
