# Supplementary material for: Extraction and purification of glomalin-related soil protein (GRSP) to determine the associated trace metal(loid)s
Source: MethodsX. 2022 Mar 18;9:101670. doi: 10.1016/j.mex.2022.101670 (PMC8983380; doi:10.1016/j.mex.2022.101670)
Supplement: Supplementary Data S1 — Supplementary Raw Research Data. This is open data under the CC BY license http://creativecommons.org/licenses/by/4.0/ [file mmc1.docx]

**Supplementary material：**

**Table S1 Background information of representative samples**

| Sample ID | Type | Location | C | N | Fe | GRSP |
| --- | --- | --- | --- | --- | --- | --- |
|  |  |  | %(w/w) | %(w/w) | %(w/w) | %(w/w) |
| 2BS103P | peat | agricultural peat soil of the Florida Everglades | 40.9 | 2.98 | 1.40 | 2.37 |
| BS102M | soil | fertile prairie soils of the US states of Indiana, Illinois, and Iowa | 2.9 | 0.25 | 2.23 | 2.10 |
| BS104L | leonardite | natural oxidation of exposed lignite from the Gascoyne Mine in Bowman County, North Dakota | 42.8 | 0.78 | 0.53 | 1.07 |
| CSS1-36 | sediment | surface sediment in mangrove patch of Danshuei River Basin, Taipei | 3.12 | 0.27 | 3.38 | 0.18 |
| CSS1-46 | sediment | surface sediment in mangrove patch of Danshuei River Basin, Taipei | 0.43 | 0.05 | 3.45 | 0.05 |
